# Influence of the Ni/Co Mass Ratio on the Microstructure and Properties of Quaternary Cu-Ni-Co-Si Alloys

**DOI:** 10.3390/ma12182855

**Published:** 2019-09-04

**Authors:** Jiang Li, Guojie Huang, Xujun Mi, Lijun Peng, Haofeng Xie, Yonglin Kang

**Affiliations:** 1 State Key laboratory of Nonferrous Metals and Processes, GRIMAT Engineering Institute Co., Ltd., Beijing 101417, China (J.L.) (G.H.) (H.X.); 2 School of Materials Science and Engineering, University of Science & Technology Beijing, Beijing 100083, China

**Keywords:** Cu-Ni-Co-Si alloy, Ni/Co mass ratio, physical properties, microstructure, atomic distribution

## Abstract

The properties and microstructural evolution of quaternary Cu-Ni-Co-Si alloys with different Ni/Co mass ratios are investigated systematically. These alloys exhibit higher mechanical properties when the Ni/Co mass ratio is 1.12-1.95 (NC-4-NC-5) and show excellent electrical conductivity when the Ni/Co mass ratio is 0.05-0.5 (NC-1-NC-3). With an increase in the Ni/Co ratio, the dimension of precipitated phase continues to increase and the grain size also visibly grows and coarsens. At the same time, the precipitation process of the NC-5 alloy is the most adequate, resulting in the highest mechanical properties. In addition, the precipitated phase in the alloys was confirmed to be the (Ni, Co)_2_Si composite phase. The number of Ni_2_Si phases in the precipitated phase gradually increased, and the Ni atoms exhibited the strongest co-segregation alongside the increasing Ni/Co ratio. Compared with the alloy without a Co element, the addition of Co helped refine the grain size and accelerate the precipitation of the particle phase and purify solute atoms in the matrix, thereby simultaneously improving mechanical properties and conductivity. The present work provides a new method for the development of multicomponent Cu-Ni-Si-Co-X alloys with outstanding comprehensive performance.

## 1. Introduction

Cu-Ni-Si alloys are widely used in electrical applications because of their high strength, excellent electric conductivity, and good anti-stress relaxation properties [1]. For the last few decades, the microstructure of the alloy has been developed by adjusting the Ni/Si ratio and optimizing the heat treatment process to further enhance the properties of the alloy [2]. Up to now, the pure ternary Cu-Ni-Si alloys has exhibited a high strength of 600–800 MPa and an electrical conductivity of 30%–45% IACS [3], but these values seem to reach the limits of ternary alloys. Therefore, the method of adding trace elements has been introduced into ternary Cu-Ni-Si alloys with the expectation that performance can be further advanced.

For instance, the addition of Al [4], Mg [5], Ti [6], Cr [7], Zr [8], V [9], or Co [10] have been proven to effectively improve the mechanical properties of Cu-Ni-Si alloys. The addition of Al and Mg can improve the precipitation kinetics and accelerate the formation rate of precipitates. Cr can significantly refine the structure by the formation of the Cr phase and more stable Cr_3_Si particles, thereby leading to a significant strengthening effect. V facilitates the precipitation process during aging, and good comprehensive performance can be obtained. Co additions to Cu-Ni-Si alloys have also been studied and found to affect the precipitation microstructure and enhance the age hardening response. Xiao [11] confirmed that Co addition impedes the occurrence of spinodal decomposition and combines with vacancies during aging. Zhao [12] identified that Co contributes a significant precipitation strengthening effect by promoting the precipitation of Cr, Ni, and Si solute atoms and preventing the coarsening of the (Cr, Co)_2_Si particles in a Cu-Ni-Si-Co-Cr alloy. Huang [13] investigated how Co can increase the nucleation rate of the δ-(Co, Ni)_2_Si phase to improve the strength in a Cu-Ni-Co-Si-Mg alloy. We [14] also proposed that Co increases strength by forming Co_2_Si precipitates and δ-(Ni, Co)_2_Si composite phases with different morphologies in a Cu-Ni-Co-Si alloy. At present, studies on the effects of Co addition on alloys exist for quinary and multi-element alloys, but these studies are inevitably influenced by the effect of the other alloying elements. In addition, current studies only add a small amount of Co as a trace element without considering the element ratio in previous Cu-Ni-Si alloys. Therefore, the effect of Co addition on Cu-Ni-Si alloys still needs to be further studied.

According to previous reports on Cu-Ni-Si alloys, the change of the Ni/Si mass ratio shows a strong correlation with the microstructure and properties of the alloy and exhibits good comprehensive properties at 4.2 [15,16]. The same method is analogous to the Cu-Ni-Co-Si alloys. It is suspected that there is a regularity in the Ni/Co ratio of Cu-Ni-Co-Si alloys. Therefore, new Cu-Ni-Co-Si alloys with different Ni/Co mass ratios were systematically designed by replacing part of Ni with Co, with the premise of ensuring the same (Ni + Co)/Si mass ratio. The microstructural evolution of alloys with different Ni/Co ratios was investigated by using an optical microscope (OM) and transmission electron microscope (TEM). The atomic distribution and content changes in the precipitated phases of these alloys were analyzed by the quantitative three-dimensional atom probe (3DAP) technique.

## 2. Experimental

The master ingots of the studied alloys were fabricated by injection melting with high purity copper, niobium, cobalt, and silicon elements of 99.95 wt.% under the protection of an argon atmosphere. The ingots with dimensions of 200 mm × 100 mm × 20 mm were cast in an iron die. The chemical compositions of the Cu-Ni-Co-Si alloys are shown in Table 1. Subsequently, the surfaces of the cast ingots were removed 2 mm from each side and hot rolled to 2 mm in thickness at 930 °C to ensure consistent quality. The heat treatment was conducted at 1020 °C or 900 °C (NC-8 alloy) for 1 h, followed by quenching in water to room temperature. Finally, the samples were isothermally aged at 450 °C and 500 °C for various times.

Vickers hardness was measured on a WILSON VH1150 hardness tester (Chicago, IL, USA) under a weight of 5 kg and a dwelling time of 15 s. Electrical conductivity was analyzed using a Sigma 2008 digital eddy current conductivity meter (St. Louis, MI, USA). Tensile samples were machined from sheets in the longitudinal direction of the peak-aging sheets, and tensile tests were carried out at room temperature on a MTS-WD 3100 material testing system (Eden Prairie, MN, USA). Each experimental value was taken from the average of 5 tensile samples. The microstructure was examined under a Zeiss-Axio Observer A1 optical microscope (Zeiss, Jena, Germany), and the statistics of the grain size were analyzed by using the Image-Pro Plus 6.0 software. TEM specimens were prepared by mechanically grinding and dimpling thin foils to a thickness of 50~60 μm followed by double bridge electropolishing in the methanol nitrate solution with a volume ratio of 4:1. TEM and an HRTEM analysis were performed using a FEI Tecnai G^2^ F20 electron microscope (Hillsboro, OR, USA). The 3DAP experiments were conducted on a LEAP 4000 HR instrument (Cameca, Gennevilliers, France), which was operated at a pulse repetition rate of 200 kHz, a voltage pulse fraction of 20%, a temperature of 20 K and a target evaporation rate of 1% under a vacuum of 10^−10^ Pa [17]. The sample blanks with a needle-like shape of 0.5 × 0.5 × 20 mm^3^ were prepared by a combination of slicing and mechanical grinding. A two-step electropolishing procedure was used to make tips from these blanks [18]. Data reconstructions and quantitative analyses were performed with the IVAS 3.6.4 software.

## 3. Results and Discussion

### 3.1. Physical and Mechanical Properties

**Figure 1:**
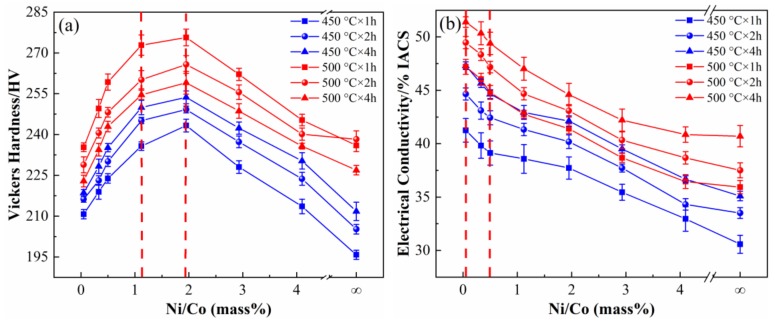
(**a**) Hardness and (**b**) electrical conductivity of alloys aged at 450 °C and 500 °C with different Ni/Co mass ratios.

Figure 1 shows the change in conductivity and hardness of Cu-Ni-Co-Si alloys with different Ni/Co mass ratios at 450 °C and 500 °C for 1, 2, and 4 h. Under the same aging conditions, the hardness and conductivity of the alloy changes in nearly the same way. As shown in Figure 1a, the hardness of the samples first rises rapidly to the peak and then decreases sharply with an increase of the Ni/Co ratios. The hardness is higher when the Ni/Co mass ratio is 1.12–1.95 (NC-4-NC-5) and reaches its peak value when the Ni/Co ratio is 1.95 (NC-5). The trend of the conductivity with the different Ni/Co ratios is shown in Figure 1b. The conductivity of the samples is higher at low Ni/Co mass ratios and decreases gradually alongside increasing Ni/Co mass ratios. The sample exhibits good conductivity with the Ni/Co ratio of 0.05–0.5 (NC-1-NC-3), and the highest conductivity is obtained at 0.05 (NC-1). Moreover, the properties of the alloy at 500 °C are much better than those at 450 °C, both in terms of conductivity and hardness.

**Figure 2:**
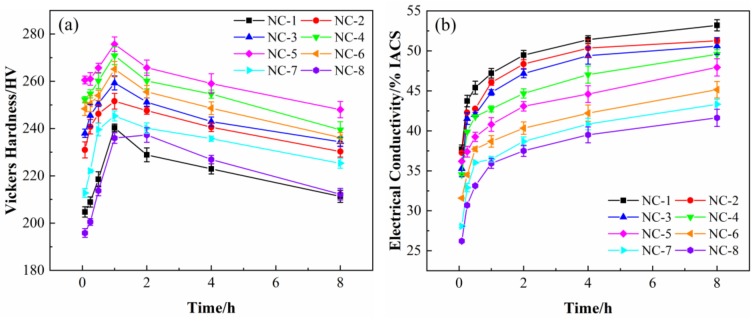
(**a**) Hardness and (**b**) electrical conductivity of alloys aged at 500 °C with different Ni/Co mass ratios.

Figure 2 shows the curves of hardness and conductivity of the Cu-Ni-Co-Si alloys aged at 500 °C for different times. As illustrated in Figure 2a, the hardness increases to the peak rapidly and then decreases slowly with an increase in aging time. It can also be seen that the alloys with different Ni/Co ratios basically reach peak aging at 500 °C for 1 h. In addition, the hardness of NC-4 and NC-5 alloys is much higher than that of other alloys with Ni/Co ratios, and the peak hardness is 270–276 HV. The conductivity of the alloy increases gradually with aging, as shown in Figure 2b. The conductivity of the NC-1-NC-3 alloy is much higher than that of the other alloys and reaches 45%–48% IACS under peak aging conditions. Because of their comprehensive properties, the NC-2, NC-4, and NC-5 alloys were selected for the follow-up comparison studies; their corresponding peak aging properties are 252 HV, 46% IACS, 270HV, 43% IACS, and 276HV, 41% IACS, respectively.

**Figure 3:**
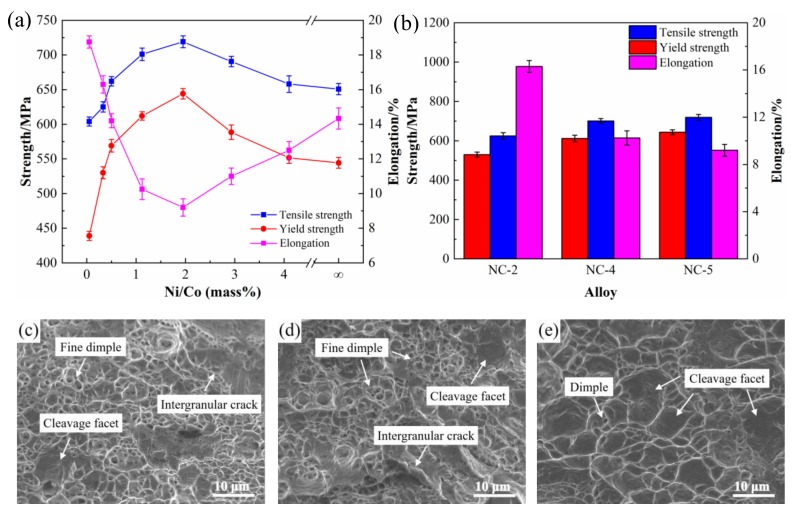
(**a**) The tensile properties of alloys aged at 500 °C for 1 h with different Ni/Co ratios; (**b**) comparison of the tensile properties of the three alloys under peak aging conditions; the corresponding fracture morphologies of the (**c**) NC-2, (**d**) NC-4, and (**e**) NC-5 alloys.

The tensile properties of the alloys with different Ni/Co ratios under peak aging conditions are shown in Figure 3a. The yield and tensile strength of the alloys show the same tendencies. The strength rapidly reaches its peak value and then gradually decreases with an increase in the Ni/Co mass ratios. On the other hand, the elongations decrease sharply and then increase slowly with an increase in the Ni/Co mass ratios. The strength and elongation of the three key alloys are compared in Figure 3b. The corresponding yield strength, tensile strength, and elongation are 530 MPa, 612 MPa, 644 MPa; 625 MPa, 701 MPa, 719 MPa, and 16.3%, 10.3%, 9.2%, respectively. Moreover, the corresponding fracture morphologies of the tensile samples are shown in Figure 3c–e. All three samples exhibit prominent cleavage crack features with a few intergranular cracks, secondary cracks, and very fine shallow flat dimples in some of the grains. Rock candy structures on the overall fracture morphology are observed, indicating typical brittle fracture characteristics. Compared with the other two alloys, the dimples of the NC-2 alloy are denser and deeper and lead to better elongation. These dimples gradually become shallower and larger with an increase on the Ni/Co ratios, which explains why the elongation gradually decreases.

### 3.2. Microstructure Observation

**Figure 4:**
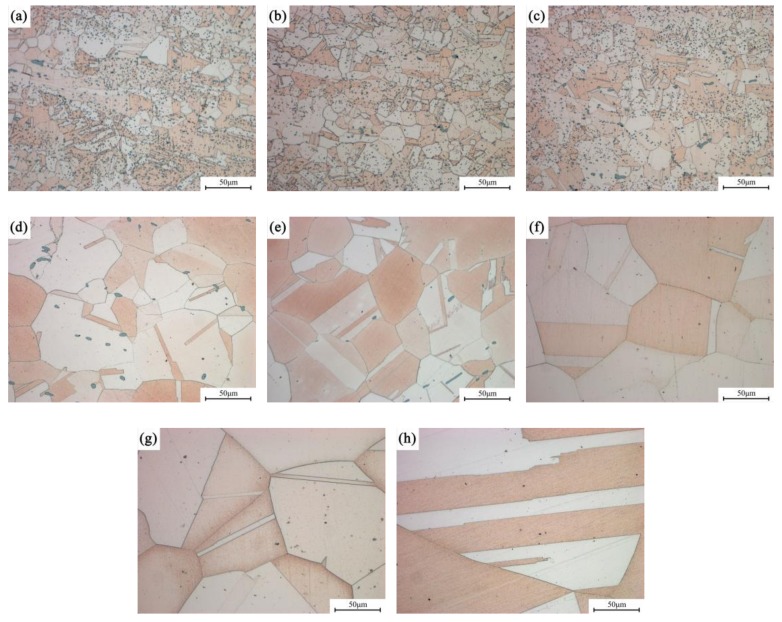
Optical microstructure of alloys aged at 1020 °C for 1 h with different Ni/Co mass ratios; (**a**–**h**) NC-1–NC-8.

Figure 4 shows the light microscope images of the Cu-Ni-Co-Si alloys with different Ni/Co mass ratios after solid solution treatment. It can be seen intuitively that the grain size in the alloy significantly increases and grows with an increase of the Ni/Co ratio. Apart from the visible grain in the alloy, a considerable number of dark particles are dispersed inside the grains and gradually decrease as the proportion increases. The preliminary analysis showed that these dark particles are Co particles that fail to dissolve into the matrix. Since the solid solubility of the Co with an hcp structure is very low in the Cu matrix with an fcc structure, the addition of a large amount of Co will inevitably lead to an insufficiently solid solution. However, it is not appropriate to improve the solid solution’s temperature or time, because the grains in a high proportion of alloys have significantly grown and coarsened.

**Figure 5:**
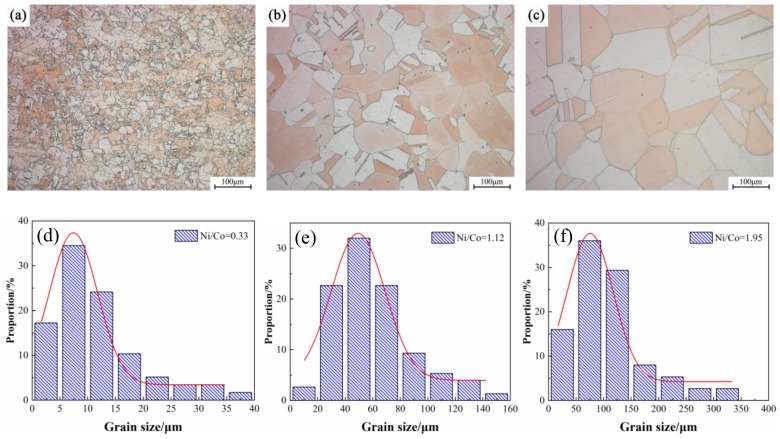
Average grain size of alloys with different Ni/Co mass ratios aged at 500 °C for 1 h; (**a**,**d**) NC-2, (**b**,**e**) NC-4, and (**c**,**f**) NC-5.

Figure 5 displays the typical optical microstructure of the alloys versus different Ni/Co mass ratios under peak aging conditions. The grain size gradually grows with an increase of the ratio, which is basically consistent with the observation of the solid solution’s state. In addition, no precipitated particles are observed in the alloy, indicating that the sizes are small at this time. The quantitative measurement results of the corresponding grain size distribution are shown in Figure 5. Compared with that of the NC-2 alloy, the average size of the NC-5 alloy increases by 10 times, and the grain size is approximately 100–125 μm. 

### 3.3. TEM Observation

**Figure 6:**
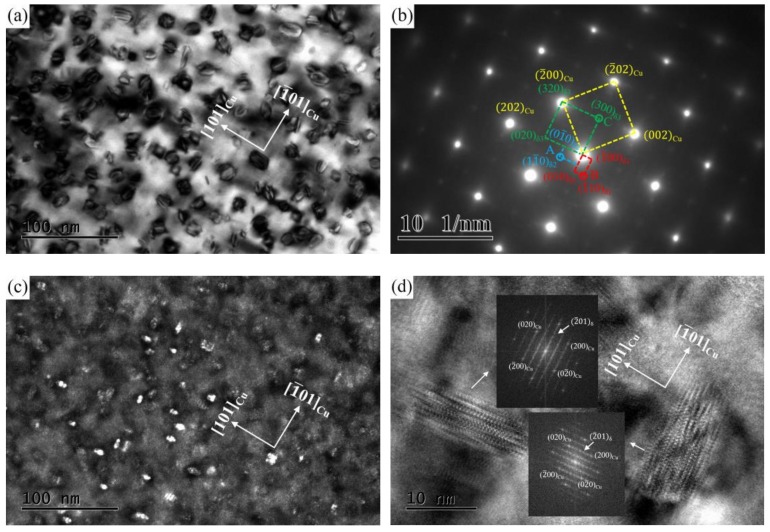
TEM images of the NC-2 alloy aged at 500 °C for 1 h; (**a**) a bright field TEM image along [001]_Cu_, (**b**) SAED corresponding of (**a**), (**c**) a dark field TEM image of A, and (**d**) an HRTEM image.

The crystallographic characteristics of the precipitated phase in the alloy at peak aging were conducted by TEM observation. Figure 6 illustrates the TEM and HRTEM images obtained from the NC-2 alloy aged at 500 °C for 1 h. Viewed from the bright field image along the [001]_Cu_ direction (Figure 6a), a large number of bean-like particle phases are uniformly dispersed in the matrix. The average size of the precipitated phase particles is approximately 6–10 nm. According to the calibration of the corresponding selected area electron diffraction (SAED) pattern (Figure 6b), three sets of precipitated phase spots appeared in the alloy in addition to the matrix spots. Two sets of diffraction spots (marked as A and B) are aligned mutually perpendicular along the <110>_Cu_ direction, and the other (marked as C) is located at the 1/2 ($\bar{2}02$)_Cu_ position. From the observation of the central dark field image (Figure 6c), the precipitated phases are perpendicular to each other and have the same orientation relationships (ORs) with the matrix, which is further confirmed by the corresponding HRTEM image (Figure 6d). Moreover, the precipitated phase and the matrix exhibit a highly coherent relationship. Combined with the above calibration and previous analysis [14], the precipitate phase in the alloy is identified to be the δ-(Ni, Co)_2_Si composite phase with an orthorhombic structure. The corresponding OR of the δ-(Ni, Co)_2_Si phase and matrix can be indexed as follows: ($\bar{2}02$)_Cu_//(010)_δ1_//(100)_δ2_//(300)_δ3_, [001]_Cu_//[100]_δ1_//[100]_δ2_//[100]_δ3_.

However, the crystallographic structure of the Ni_2_Si, Co_2_Si, and (Ni, Co)_2_Si phases have orthorhombic structures, and their lattice constants are a = 0.71 nm, b = 0.50 nm, c = 0.37 nm, and a = 0.71 nm, b = 0.49 nm, c = 0.37 nm, and a = 0.71 nm, b = 0.50 nm, c = 0.37 nm, respectively [19,20]. Since the three precipitated phases have the same lattice constants and crystal structures, it is difficult to distinguish them by TEM observation. Therefore, the distribution of the precipitated phases will be further analyzed by the 3DAP technique.

**Figure 7:**
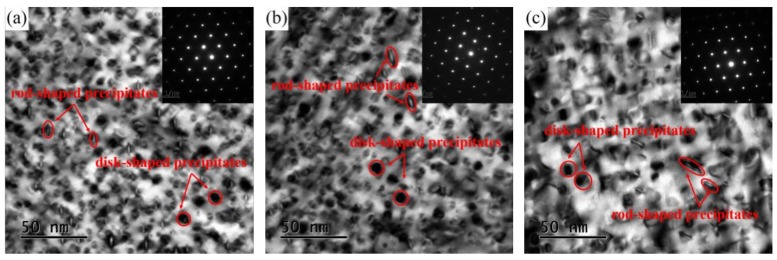

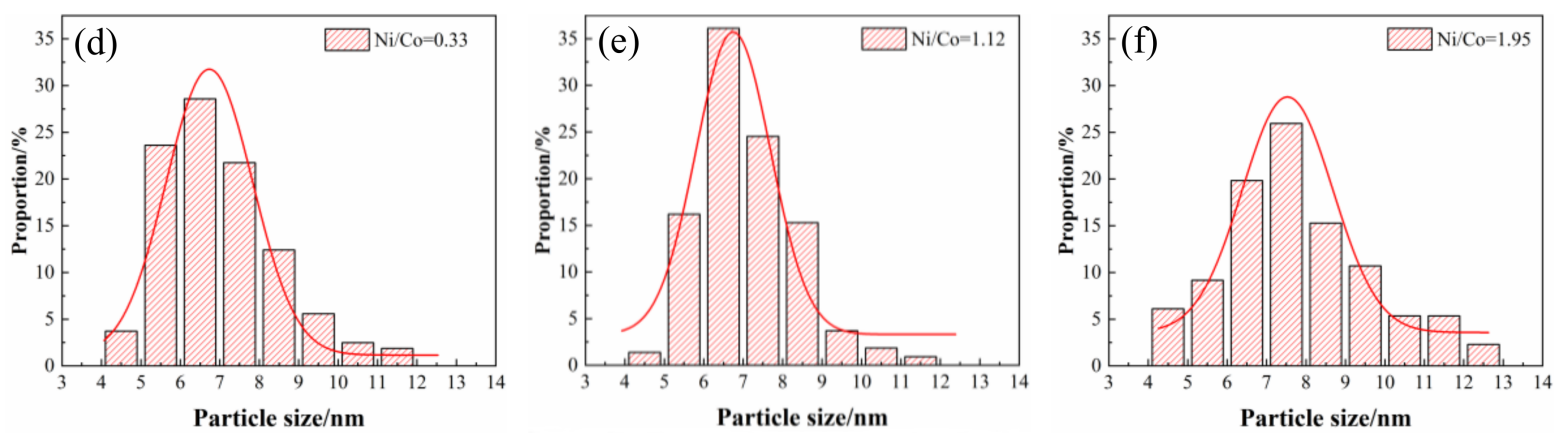
TEM images along [110]_Cu_ and the particle size of alloys with different Ni/Co mass ratios aged at 500 °C for 1 h; (**a**,**d**) NC-2, (**b**,**e**) NC-4, and (**c**,**f**) NC-5.

Figure 7 illustrates the bright field TEM images of the alloys with different Ni/Co mass ratios along the [001]_Cu_ direction. A large number of precipitated particles are formed and dispersed in the matrix. In addition, the different disk- and rod-like shape characteristic are clearly visible in each bright field image. Based on the energy minimum principle, a precipitate preferentially grows along the direction of the lowest energy. The different morphologies of the precipitates are mainly due to their different growth directions, which were also reported in previous studies [3,21]. With an increase in the Ni/Co ratio, the dimension of the precipitated phase gradually increases, and the distribution becomes denser. The corresponding quantitative statistical results of the size distribution are shown in Figure 7d–f. The variation range of the average particle size remained around 6–9 nm, and the alloys are 6.98 nm, 7.96 nm, and 8.76 nm, respectively. In addition, the volume fraction and the number density of the precipitated phase also increased continuously, indicating that the precipitation process is more adequate. The main reason for this phenomenon is that many particles are not dissolved into the matrix at a low Ni/Co ratio, and the solid solution is insufficient, resulting in the lack of a precipitation driving force in the subsequent precipitation process [22]. 

### 3.4. 3DAP Analysis 

**Figure 8:**
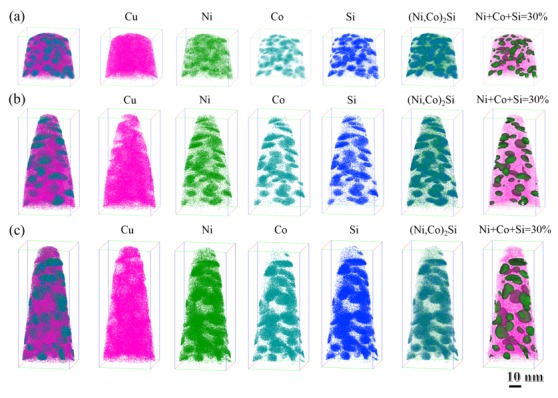
Three-dimensional atom probe (3DAP) elemental maps showing Cu, Ni, Co, and Si atoms and the corresponding map of precipitates in the studied alloys aged at 500 °C for 1h; (**a**) NC-2, (**b**) NC-4, and (**c**) NC-5.

The 3DAP maps of the atomic distribution for alloys with different Ni/Co mass ratios are given in Figure 8. The 3DAP elemental maps show the Cu, Ni, Co, and Si atoms individually, as well as a map of the precipitated phase δ-(Ni, Co)_2_Si (Cu atoms have been excluded). Several regions where the Ni, Co, and Si atoms have co-segregated are clearly visible, while Cu atoms are randomly distributed in the alloy. The Ni, Co, and Si rich regions display a roughly bean-like morphology, which is consistent with previous TEM observations. In addition, it can be clearly seen that no matter how the Ni/Co ratio changes, the second phase exists in the form of the (Ni, Co)_2_Si phase instead of the Ni_2_Si or Co_2_Si phase. In order to better explore the changes in the internal atoms of the alloys, the samples are divided into iso-concentration surfaces containing 30 at.% (Ni, Co, and Si), as shown in Figure 8. The green areas represent the collected precipitated phases, and the pink areas are Cu atoms. The tendency of the precipitated phase size and volume fraction to increase with an increase in the Ni/Co ratio is readily observed.

**Figure 9:**
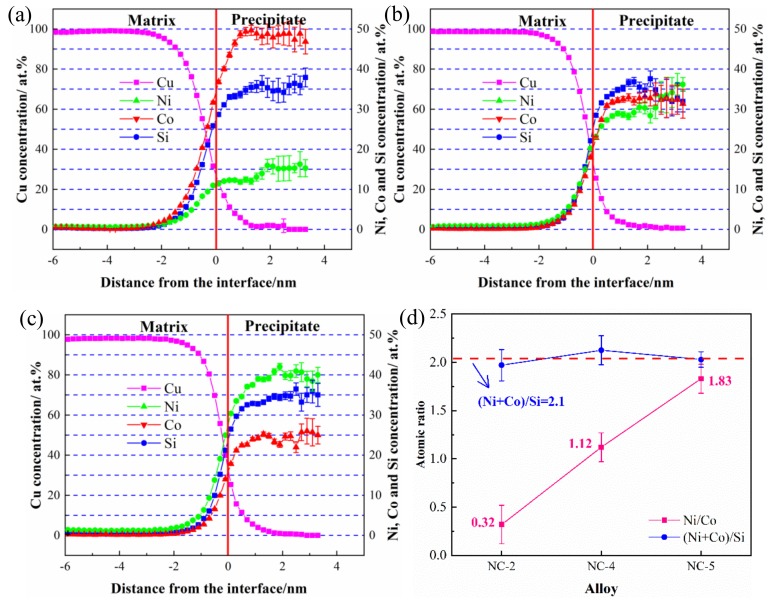
Atomic concentration distribution profiles of the studied alloy aged at 500 °C for 1 h; (**a**) NC-2, (**b**) NC-4, (**c**) NC-5, and (**d**) variations of the Ni/Co and (Ni + Co)/Si atomic ratios.

The corresponding atomic concentration distribution of all collected precipitates identified is plotted as a function of distance in Figure 9a–c. The red line indicates the phase interface between the substrate and the precipitate phase, and the concentration changes of the Cu matrix and the solute atoms correspond to the left and right sides of the line, respectively [23]. The variation of atomic concentration shows the same tendency in the three alloys. The content of solute atoms continues to increase until it reaches equilibrium, while Cu atoms, with an increase in distance, decrease until they disappear. After reaching a saturated state in Figure 9a, the Ni, Co, and Si atom contents in the precipitated phase of the NC-2 alloy are 15%, 50%, and 35%, respectively. After increasing the Ni/Co mass ratio to 1.12 (Figure 9b), the concentration of the Ni, Co, and Si atoms in the NC-4 alloy are 36%, 32%, and 32%, respectively. The concentration of solute atoms in the NC-5 alloy are 42%, 23%, and 35%, respectively (Figure 9c). In this process, the content of Ni increases significantly, and Si remains unchanged in the precipitated phase, while the content of Co decreases continuously. To some extent, it is reasonable to believe that the atomic content of Ni and Co in the alloy represents the proportion of Ni_2_Si and Co_2_Si in the precipitated phase [24]. Moreover, the average Ni/Co and (Ni + Co)/Si atomic ratios of the three alloys are calculated in Figure 9d. The (Ni + Co)/Si atomic ratios remain approximately 2.1, and the corresponding mass ratios are 4.2, indicating that the precipitated phase is (Ni, Co)_2_Si. Since Ni and Co have similar relative atomic masses (58.69 and 58.93), their atomic ratios can be considered approximately equal to their mass ratios [25]. Hence, the Ni/Co and (Ni + Co)/Si atomic ratios in the precipitated phase are highly consistent with the designed contents. 

**Figure 10:**
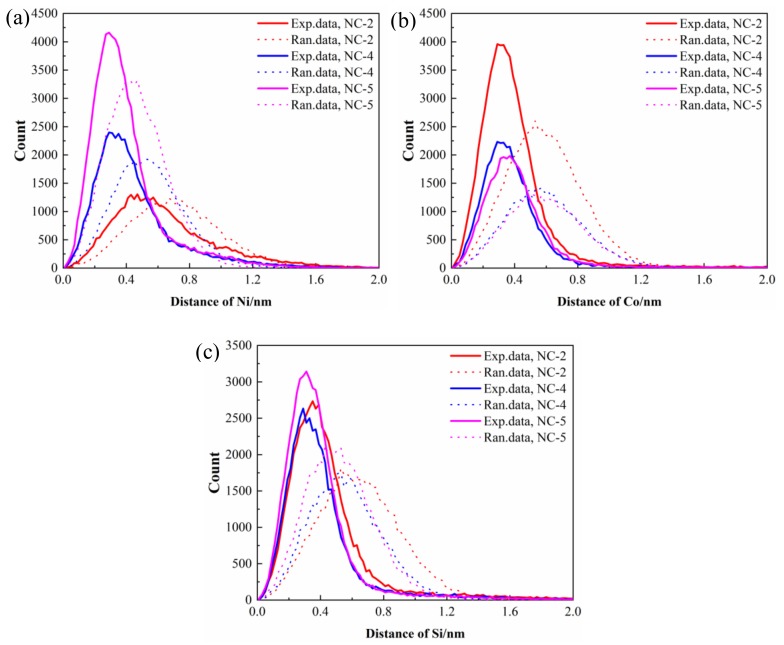
The first-nearest-neighbor distribution (1NND) curves (experimental data shown as solid lines and randomized data shown as dotted lines) of solute atoms in the studied alloy aged at 500 °C for 1 h; (**a**) Ni, (**b**) Co, and (**c**) Si.

Figure 10 illustrates the corresponding first nearest neighbor (1NN) distribution analysis of solute atoms for all studied alloys. The distance between the experimental value and standard normal distribution curve represents the segregation of solute atoms in the precipitated phase [26]. Among the three alloys, Ni atoms of the NC-5 alloy and Co atoms of the NC-2 alloy show the most serious co-segregation, which corresponds well with their atomic content. For example, the amount of the Co_2_Si phase formed is much more than that of the Ni_2_Si phase in the NC-2 alloy, which leads to the larger 1NN distances of Co atoms compared to those of other solute atoms. Compared to Ni and Co atoms, the experimental 1NN distribution of Si atoms in the three alloys deviates slightly from the 1NN distributions of the corresponding random data.

### 3.5. Microstructure—Properties Relationship

#### 3.5.1. Mechanical Properties

There are four main strengthening mechanisms for the Cu-Ni-Co-Si alloy, solid solution strengthening, grain boundary strengthening, precipitation strengthening, and dislocation strengthening, respectively [27]:

(1) $$\sigma_{total}=\sigma_{0}+\Delta \sigma_{GB}+\Delta \sigma_{p}+\Delta \sigma_{s}+\Delta \sigma_{d}.$$

Solid solution strengthening is mainly attributed to the lattice distortion arising from different solute atoms, while the solute atoms in the Cu-Ni-Co-Si alloy are Ni, Co, and Si atoms. According to the previous 3DAP analysis, the solid solution atoms are almost precipitated in the form of the (Ni, Co)_2_Si phase in the peak aging state. Therefore, the strengthening effect of the residual solid solution atoms in the matrix can be ignored. In addition, dislocation strengthening mainly comes from the dislocation entanglements generated during cold deformation [28]. The cold deformation process is not introduced in this study, so the dislocation strengthening effect is also neglected. Therefore, the effect of Ni/Co mass ratios on strength is mainly due to precipitation strengthening and grain boundary strengthening.

In general, precipitation strengthening is the dominant strengthening method for age-strengthened alloys, which can be divided into the cutting mechanism and bypass mechanism [29]. The main mechanism for the Cu-Ni-Co-Si alloy is the bypass mechanism, which can be expressed as the Orowan dislocation bypass mechanism [30]:

(2) Δσp=0.81MGb2π1−ϑ12lndp/bdp3π8fv−1,

where *M* is the Taylor factor, *G* is the shear modulus of the matrix, *d_p_* is the average diameter of the particles, *b* is the Burgers vector, *ν* is the Poisson’s ratio, and *f_v_* is the volume fraction of the precipitated phase. The increment of stress *Δσ_p_* is inversely proportional to the average diameter *d_p_* but proportional to the volume fraction *f_v_*. 

**Figure 11:**
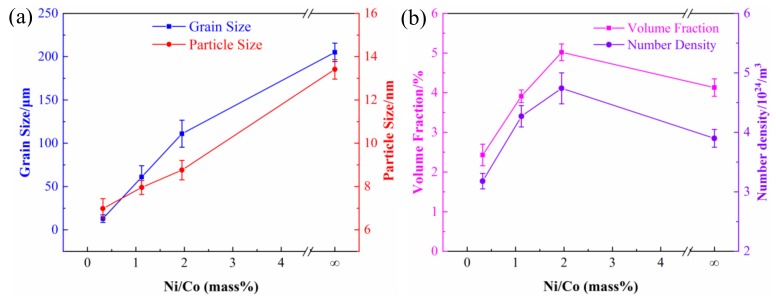
Variation curves of (**a**) the average grain and particle size and (**b**) the volume fraction and number density of alloys with different Ni/Co mass ratios.

Figure 11a shows the variation of the average grain and particle sizes of the alloys with different Ni/Co mass ratios as well as quantitative data for the NC-8 alloy (Ni/Co = ∞) from previous research results [3]. The average grain and particle size of the precipitated phase show the same trend of change, as both gradually increase with an increase of the Ni/Co mass ratios. Except for the NC-8 alloy, the average grain size of the other three alloys is generally maintained at approximately 1–3 nm. Figure 11b presents the change in the volume fraction and quantity density of the precipitates. It can be clearly observed that the volume fraction of the NC-5 alloy is much higher than that of the other alloys, leading to its excellent hardness and strength. The variation of volume fraction is basically consistent with that of hardness and strength, indicating that the volume fraction plays a decisive role for these three alloys. In addition, it can be found that the number density of the NC-5 alloy is also the largest, which further illustrates that the precipitation process is more adequate. At low Ni/Co mass ratios, the solid solution process is insufficient, resulting in an insufficient precipitation driving force in the aging process. Consequently, the number density is much lower than that of the high Ni/Co ratios.

The grain effectively blocks the dislocation movement and strengthens the grain boundary, which can be expressed by the Hall–Petch equation [31,32]:

(3) $$\Delta \sigma_{GB}=K_{y}d_{g}^{-1/2},$$

where *K_y_* is the Hall–Petch coefficient, and *d_g_* is the average grain diameter. Based on the formula, the square of grain size *d_g_* is inversely proportional to the strength increment *Δσ_GB_* [33]. The corresponding grain size changes are shown in Figure 11a. The grain size gradually increases as the Ni/Co mass ratio increases, resulting in a gradual decrease in grain boundary strengthening. When the proportion increases, the grain boundary strengthening is gradually weakened with the growth of the grain size.

According to the above analysis, the NC-5 alloy exhibits excellent mechanical properties with a combination of grain boundary strengthening and precipitation strengthening. Compared to the NC-8 alloy without Co, the addition of Co refines the size of precipitates and grains. Compared to other ratio alloys, the precipitation process can be fully carried out to form (Ni, Co)_2_Si phases without coarsening grain sizes when the Ni/Co mass ratio is 1.12–1.95. 

#### 3.5.2. Conductive Property

The conductive mechanism for the Cu-Ni-Co-Si alloy is mainly derived from the following contributions, which can be described by the Mattiessen relation [34]:

(4) $$\rho_{total}=\rho_{0}+\Delta \rho_{GB}+\Delta \rho_{p}+\Delta \rho_{s}+\Delta \rho_{d},$$

where $\rho_{0}$ is the electrical resistivity of pure copper, and $\Delta \rho_{GB},\;\Delta \rho_{p},\;\Delta \rho_{s}$, and $\;\Delta \rho_{d}$ are the electrical resistivity increments arising from the grain boundary, precipitation, solute atoms, and dislocation scattering, respectively. The average grain size is coarsened and the dislocation density is moderately low in the alloy, so the effects of these factors can be ignored. Moreover, the second phase precipitated from the alloy has little effect on electrical conductivity [35]. Hence, $\Delta \rho_{s}$ is the most important factor affecting electrical conductivity, which depends mainly on the amount of solid solution atoms in the matrix. The increase in the number of solid solution atoms in the matrix leads to an increase in the lattice distortion of the matrix, which improves the scattering of electrons and affects the conductivity of the alloy.

At a low Ni/Co mass ratio, the Co atoms in the matrix are more numerous than Ni atoms. With the aging process, the Co and Si atoms in the matrix precipitate out in the form of the Co_2_Si phase. In addition, since the Co atom is an hcp structure, it is poorly compatible with the Cu matrix of the fcc structure, thereby further accelerating the precipitation of the precipitated phase [36]. During this process, the number of solid solution atoms in the matrix is significantly reduced, resulting in higher conductivity. When the Ni/Co mass ratio is about 1.12–1.95, Since the binding energy of the Co and Si atoms is greater than that of the Ni and Si atoms, Co preferentially combines with Si to form Co_2_Si phases [37]. However, the low content of Co atoms in the alloy is not enough to exhaust all Si atoms, resulting in the formation of Ni_2_Si phases at the same time. As the ratio increases, the precipitated phases are mainly Ni_2_Si phases at a high Ni/Co ratio. A portion of the Ni atoms remains in the matrix because the Ni atoms with the same fcc structure can be better dissolved into the matrix Cu atoms. This increases the lattice distortion of the matrix and enhances the scattering of electrons, resulting in decreased conductivity.

Combined with the previous analysis of mechanical properties, the alloy exhibits great precipitation kinetics and grain refinement when the Ni/Co mass ratio is around 1.12-1.95 (NC-4-NC-5). The precipitating extent of the second phase is sufficient and the contents of Ni, Co, and Si atoms in the matrix are substantially reduced to form (Ni, Co)_2_Si precipitates. Furthermore, the strain energy is effectively reduced, and the matrix is further purified, resulting in remarkable improvement of electrical conductivity. Therefore, good comprehensive performance with a combination of conductivity and mechanical properties can be obtained. 

## 4. Conclusions

The variation of the properties and microstructure of Cu-Ni-Co-Si alloys shows a strong correlation with different Ni/Co mass ratios. With an increase of the Ni/Co ratio, the hardness and strength of the alloy increases rapidly to the peak and then decreases sharply, while the conductivity shows a gradually decreasing trend. The alloys exhibit higher mechanical properties when the Ni/Co mass ratio is 1.12–1.95 (NC-4-NC-5) and show excellent electrical conductivity when the Ni/Co mass ratio is 0.05–0.5 (NC-1-NC-3). In addition, the highest volume fraction and number density of the precipitated phase are obtained when the Ni/Co ratio is 1.95. The precipitated particle in the alloys was identified as the (Ni, Co)_2_Si composite phase, and the amount of the Ni_2_Si phase gradually increased with an increase of the Ni/Co ratio. Theoretical analysis shows that the high strength of alloys with different Ni/Co ratios is mainly due to precipitation strengthening and secondarily attributed to grain boundary strengthening. The addition of Co can help to refine grain size and improve the solid solution temperature of an alloy. Moreover, it can accelerate the precipitation of the second phase and purify solute atoms in the matrix, thereby resulting in high strength and excellent electrical conductivity. 

## Figures and Tables

**Table 1:** Designed and tested chemical compositions of Cu-Ni-Co-Si alloys, wt.%.

Alloy	Cu	Ni	Co	Si	Ni/Co	(Ni + Co)/Si	Ni + Co + Si
NC-1	Bal.	0.17	3.36	0.84	0.05	4.2	4.3
NC-2	Bal.	0.85	2.67	0.82	0.32	4.3	4.3
NC-3	Bal.	1.16	2.30	0.82	0.50	4.2	4.3
NC-4	Bal.	1.84	1.64	0.82	1.12	4.2	4.3
NC-5	Bal.	2.31	1.18	0.82	1.95	4.3	4.3
NC-6	Bal.	2.60	0.89	0.82	2.92	4.3	4.3
NC-7	Bal.	2.80	0.68	0.82	4.12	4.3	4.3
NC-8	Bal.	3.48	-	0.82	∞	4.2	4.3
